# Limited Generalizability of Registration Trials in Hepatitis C: A Nationwide Cohort Study

**DOI:** 10.1371/journal.pone.0161821

**Published:** 2016-09-06

**Authors:** Floor A. C. Berden, Robert J. de Knegt, Hans Blokzijl, Sjoerd D. Kuiken, Karel J. L. van Erpecum, Sophie B. Willemse, Jan den Hollander, Marit G. A. van Vonderen, Pieter Friederich, Bart van Hoek, Carin M. J. van Nieuwkerk, Joost P. H. Drenth, Wietske Kievit

**Affiliations:** 1 Department of Gastroenterology and Hepatology, Radboud university medical center, Nijmegen, the Netherlands; 2 Department of Gastroenterology and Hepatology, Erasmus Medical Center Rotterdam, Rotterdam, the Netherlands; 3 Department of Gastroenterology and Hepatology, University medical center Groningen, Groningen, the Netherlands; 4 Department of Gastroenterology and Hepatology, Onze Lieve Vrouwe Gasthuis, location West, Amsterdam, the Netherlands; 5 Department of Gastroenterology and Hepatology, University medical center Utrecht, Utrecht, the Netherlands; 6 Department of Gastroenterology and Hepatology, Academic Medical Center, Amsterdam, the Netherlands; 7 Department of Internal Medicine, Maasstad hospital, Rotterdam, the Netherlands; 8 Department of Internal Medicine, Medical center Leeuwarden, Leeuwarden, the Netherlands; 9 Department of Gastroenterology and Hepatology, Catharina hospital, Eindhoven, the Netherlands; 10 Department of Gastroenterology and Hepatology, Leiden University Medical Center, Leiden, the Netherlands; 11 Department of Gastroenterology and Hepatology, VU university medical center, Amsterdam, the Netherlands; 12 Department for Health Evidence, Radboud university medical center, Nijmegen, the Netherlands; Chiba University, Graduate School of Medicine, JAPAN

## Abstract

**Background:**

Approval of drugs in chronic hepatitis C is supported by registration trials. These trials might have limited generalizability through use of strict eligibility criteria. We compared effectiveness and safety of real world hepatitis C patients eligible and ineligible for registration trials.

**Methods:**

We performed a nationwide, multicenter, retrospective cohort study of chronic hepatitis C patients treated in the real world. We applied a combined set of inclusion and exclusion criteria of registration trials to our cohort to determine eligibility. We compared effectiveness and safety in eligible vs. ineligible patients, and performed sensitivity analyses with strict criteria. Further, we used log binomial regression to assess relative risks of criteria on outcomes.

**Results:**

In this cohort (n = 467) 47% of patients would have been ineligible for registration trials. Main exclusion criteria were related to hepatic decompensation and co-morbidity (cardiac disease, anemia, malignancy and neutropenia), and were associated with an increased risk for serious adverse events (RR 1.45–2.31). Ineligible patients developed significantly more serious adverse events than eligible patients (27% vs. 11%, p< 0.001). Effectiveness was decreased if strict criteria were used.

**Conclusions:**

Nearly half of real world hepatitis C patients would have been excluded from registration trials, and these patients are at increased risk to develop serious adverse events. Hepatic decompensation and co-morbidity were important exclusion criteria, and were related to toxicity. Therefore, new drugs should also be studied in these patients, to genuinely assess benefits and risk of therapy in the real world population.

## Introduction

Regulatory approval of drugs and the development of guidelines are supported by evidence generated by registration trials. These trials aim for high internal validity through use of strict eligibility criteria, although this may jeopardize generalizability. [1, 2] Some studies suggest that many real world patients would be excluded from registration trials and that drugs tested through these trials are less effective or less well tolerated in these patients.[3–5]

The treatment arsenal for chronic hepatitis C patients (CHC) has increased enormously with the introduction of Direct Acting Antivirals (DAAs). DAAs were approved by regulatory authorities for use in clinical practice, with evidence coming from registration trials having strict criteria.[6] Indeed, real world cohorts contain large number of treated CHC patients who would be excluded from registration trials.[7–10] A lack of generalizability is only an issue when ineligible patients have worse outcomes, but this is not known for CHC. We hypothesize that CHC patients ineligible for trials, but who are treated in clinical practice have characteristics that are risk factors for treatment failure and toxicity.

Therefore, we aim to compare effectiveness and safety in real world CHC patients who are eligible or ineligible for registration trials. Our secondary aim is to identify criteria that impact trial eligibility and assess the risk of these criteria on outcomes.

## Materials and Methods

### Population and design

We conducted a nationwide, multicenter, retrospective real world cohort study of CHC patients in the Netherlands. We chose genotype 1 patients treated between 2011 and 2015 with telaprevir or boceprevir with peg-interferon and ribavirin as an example cohort. We identified CHC patients using up-to-date local databases. Treatment indication, choice of therapy, drug dosing and duration were at the discretion of the physician, following national guidelines. [11] Patients co-infected with HIV or hepatitis B virus (HBV) were excluded.

Formal evaluation was waived by the institute review board Committee on Research Involving Human Subjects Arnhem-Nijmegen given the retrospective character of our study. However, approval in participating centers was obtained according to local regulations. The study was conducted in accordance with good clinical practice guidelines and the code of conduct for medical research (www.federa.org). We obtained oral informed consent or collected data anonymously in accordance with the code of conduct for medical research. No identifying patient data was collected, and all patient data was anonymously entered in the database.

### Identification of registration trials and general set of eligibility criteria

We identified registration trials of telaprevir and boceprevir in CHC patients through a systematic search (S1 Table). We extracted eligibility criteria from published protocols, and used the least stringent criteria of all studies to develop a general criteria set (Table 1). We applied the general set to our real world population to determine eligibility. If variables were missing, we assumed the patient would be eligible for that criterion.

**Table 1:** Set of general eligibility criteria.

Variable	Criterion
**Inclusion**	
Age	Subject ≥ 18 years
Hepatitis C virus (HCV) RNA	HCV RNA detectable
Weight	Weight between 40–125 kg
Hepatocellular Carcinoma (HCC)	Ultrasound with no signs of HCC
**Exclusion**	
Genotype HCV	HCV with > 1 subtype or genotype
Hemoglobin	Hemoglobin <12 g/dL for females or <13 g/dL for males
Neutrophil count	Absolute neutrophil count <1.2 x10^9^/L
Platelet count	Platelet count <90 x10^9^/L
Albumin	Serum albumin < 3.3 g/dL
Bilirubin	Total bilirubin > 1.8x ULN†
International Normalized Ratio (INR)	INR ≥ 1.5
Thyroid Stimulating Hormone (TSH)	TSH > 1.2 x ULN or 0.8x LLN†
Alanine aminotransferase (ALT)	ALT 10 x ULN†
Aspartate aminotransferase (AST)	AST 10 x ULN†
Contra-indication to peginterferon or ribavirin	
• Hemoglobinopathy • Cardiac disease • Renal insufficiency	• Hemoglobinopathy present (thallassemia major, sickle-cell disease) • Significant cardiac disease present^a^ • Creatinine clearance ≤ 50 ml/min
Auto-immune disease	Presence of auto-immune disease^b^
Pulmonary disease	History of chronic pulmonary disease with impairment (COPD gold III or IV, interstitial lung disease, pulmonary fibrosis or sarcoidosis)
Current or history of decompensated liver disease	Current or history of ascites, encephalopathy or bleeding varices
Other liver disease	Presence of another liver disease
Malignancy	Active malignant disease or malignant disease in past 5 years (except basal cell carcinoma)
Pancreatitis	History of acute pancreatitis in past 5 years
Retinopathy	Presence of retinopathy
Seizure	Presence of a seizure disorder requiring medication
Transplantation	Patient with a history of an organ transplant
Psychiatric comorbidity	Presence of severe psychiatric disease^c^
Corticosteroids	Use of systemic corticosteroids
Hemophilia	Hemophilia present
Central nervous system (CNS) disorder	CNS disorder present^d^
Malabsorption	History of malabsorption disorder
Indwelling cathether	Subject with indwelling venous catheter
**Comedication**	Prohibited comedication listed in protocols

^a^ Significant cardiac disease was defined as: current or history of unstable cardiac disease (angina, congestive heart failure, recent myocardial infarction, pulmonary hypertension, complex congenital heart disease, cardiomyopathy, and/or significant arrhythmia)

^b^Auto-immune disease was defined as: immunologically mediated disease (inflammatory bowel disease, celiac disease, rheumatoid arthritis, idiopathic thrombocytopenic purpura, systemic lupus erythematosus, autoimmune hemolytic anemia, scleroderma, sarcoidosis, severe psoriasis, or autoimmune hepatitis)

^c^ Psychiatric comorbidity was defined as: severe depression or hospitalization for depression, schizophrenia, bipolar illness, severe anxiety or personality disorder, a period of disability or impairment due to a psychiatric disease within the past 5 years

^d^ CNS disorder was defined as: CNS trauma requiring intubation, intracranial pressure monitoring, brain meningeal/skull surgery, or resulting in seizure, coma, neurologic deficits, abnormal brain imaging, cerebrospinal fluid leak, prior brain hemorrhage and/or intracranial aneurysms, or history of stroke or transient ischemic attack

† ULN = upper limit of normal; LLN = lower limit of normal

### Data acquisition and definitions

We extracted demographics, CHC characteristics, and laboratory values from the patients’ medical records on a pre-designed case report form. Baseline variables were collected at the start of treatment not exceeding one year prior to treatment. Baseline concomitant medication was collected prior to possible medication switch for expected interactions. Data was collected until 24 weeks after cessation of treatment. We collected whether patients had a history of or current decompensated liver disease, this was defined as a history or signs of ascites, variceal bleed or hepatic encephalopathy. Effectiveness was defined as sustained virological response (SVR): undetectable hepatitis C virus RNA 12 or 24 weeks after cessation of treatment. Safety data included adverse events (AEs) and serious adverse events (SAEs). AEs were defined as any event that required 1) dose reduction of peg-interferon or ribavirin, 2) prescription of medication or 3) referral. We used the FDA definition for SAEs.[12] We categorized AEs and SAEs by common terminology criteria for adverse events (CTCAE version 4.0).[13] We recorded data anonymously in an Access database (Microsoft Access 2007).

### Outcomes and analysis

The primary outcomes were SVR and (S)AE rates, which were compared between patients eligible and ineligible for registration trials. Furthermore, we identified criteria that affected eligibility and were associated with the outcomes. Analyses were performed on an intention to treat population, where telaprevir and boceprevir treated patients were pooled. To check validity of pooling, we compared baseline characteristics and treatment outcomes between telaprevir and boceprevir patients.[14]

SVR rates, and (S)AE rates were analyzed with χ^2^ (or Fisher exact if counts <5), and Mann-Whitney U test (median number of (S)AEs). For analyses on SVR, we separated patients into two groups based on expected similar effectiveness: 1) treatment-naive and relapse patients, and 2) patients with a prior non-response, viral breakthrough or early discontinuation [15]; for safety outcomes this distinction was not made. We used frequency counts to identify most important eligibility criteria. To study the association of criteria and outcomes, we performed log binomial regression (relative risk) or poisson regression.[16] To explore the validity of our generated set of the least stringent criteria from the protocols, we performed three sensitivity analyses: a) with most stringent criteria (S2 Table), b) with strictest exclusion of co-morbidity, and c) with the most important factor for exclusion eliminated from the criteria set. All analyses were two-sided with a significance level of p <0.05, and performed in SPSS (IBM SPSS Statistics 20).

## Results

### Population

**Fig 1:**
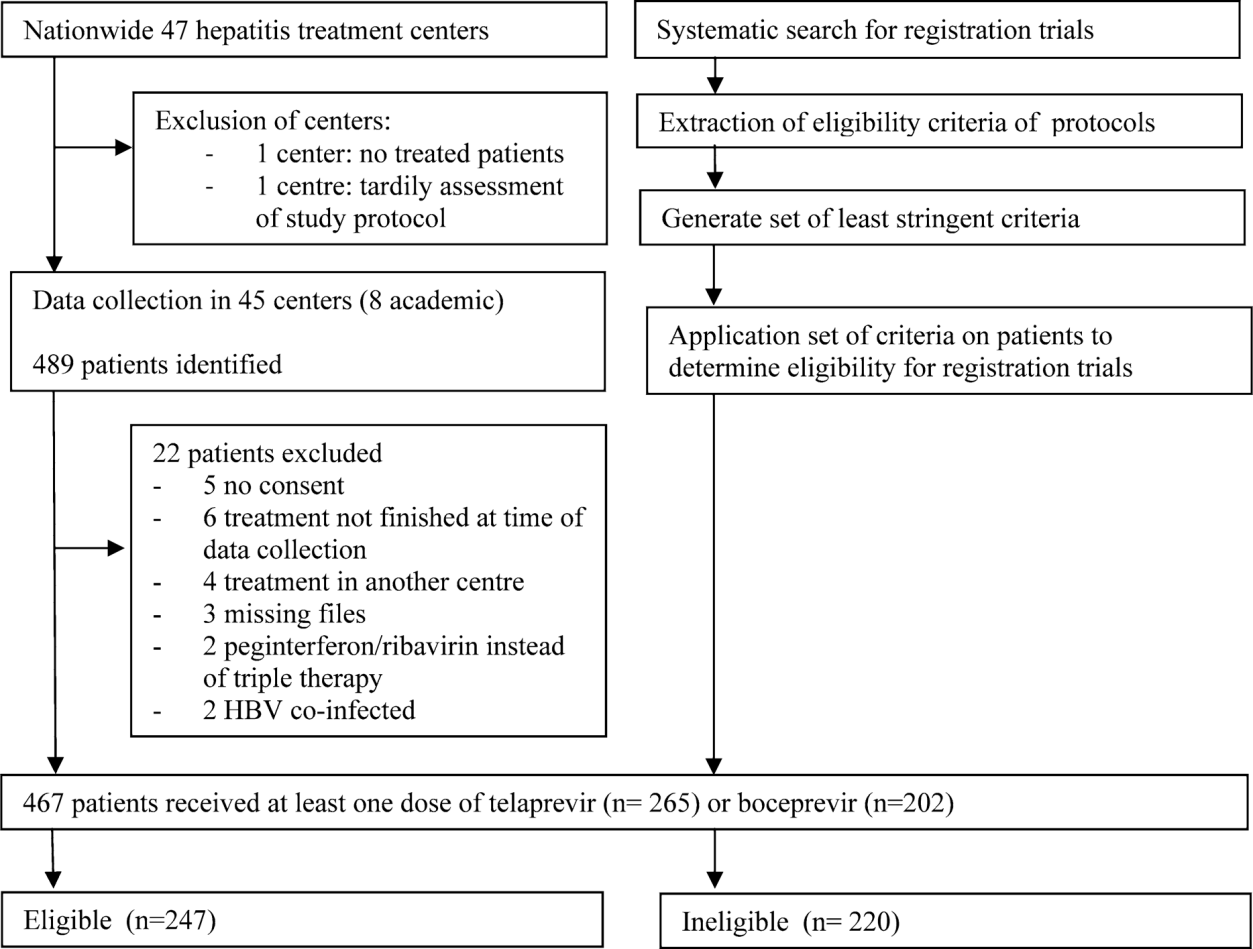
Study flowchart. The flowchart shows both enrollment of patients in all centers and assessment of eligibility for registration trials in this study.

We identified 489 treated patients from 45 centers, and we excluded 22 patients (Fig 1). Centers treated a median of 8 patients (range 1–53). Overall, the majority of patients (60%) was treatment naive, 52% had advanced fibrosis or cirrhosis and 5% had a history of decompensated liver disease. Baseline characteristics are shown in Table 2. We pooled telaprevir (n = 265) and boceprevir (n = 202) data, as there were no significant differences in characteristics and treatment outcomes between patients (S3 and S4 Tables).

**Table 2:** Baseline characteristics.

Characteristic	Overall (n = 467)	Eligible (n = 247)	Ineligible (n = 220)	p-value
Age, y–mean (range)	51 (19–77)	50 (22–77)	52 (19–70)	0.07
Male sex–n (%)	319 (68)	170 (69)	149 (68)	0.80
White race–n (%)^a^	321 (89)	173 (91)	148 (88)	0.08
HCV genotype–n (%)				0.23
• Genotype 1 indeterminate • Genotype 1a • Genotype 1b	• 86 (18) • 226 (48) • 155 (33)	• 49 (20) • 122 (49) • 76 (31)	• 37 (17) • 104 (47) • 79 (36)	
Previous response^b^				0.81
• Naive • Relapse • Nonresponse • Viral breakthrough • Early discontinuation	• 273 (60) • 76 (17) • 78 (17) • 16 (4) • 11 (2)	• 142 (59) • 45 (19) • 41 (17) • 9 (4) • 5 (2)	• 131 (62) • 31 (15) • 37 (18) • 7 (3) • 6 (3)	
Current or history of decompensated liver disease–n (%)	24 (5)	0 (0)	24 (11)	<0.001
Metavir score F3-4^c^	161 (52)	66 (42)	95 (63)	<0.001
Laboratory values^d^				
Haemoglobin g/dL—mean (SD)	9.1 (0.9)	9.2 (0.8)	9.0 (1.0)	0.02
Leucocyte count x10^9^/L—mean (SD)	6.7 (2.2)	7.0 (2.1)	6.4 (2.2)	0.003
Neutrophil count x10^9^/L—mean (SD)	3.5 (1.5)	3.6 (1.5)	3.3 (1.5)	0.22
Platelet count x10^9^/L–mean (range)	192 (24–764)	207 (90–388)	175 (24–764)	<0.001
Albumin g/dL–mean (range)	4.1 (2.4–5.1)	4.3 (3.3–5.1)	4.0 (2.4–5.1)	<0.001
Total bilirubin g/dL–median (IQR)	10.0 (7–14)	9 (7–13)	11 (8–16)	<0.001
Child Pugh (CP) score^e^				0.001
• A–n (%) • B–n (%) • C–n (%)	• 212 (95) • 11 (5) • 0 (0)	• 107 (100) • 0 (0) • 0 (0)	• 105 (91) • 11 (10) • 0 (0)	

^a^ Race: available in 360 patients;

^b^ Previous response: available in 454 patients;

^c^ Metavir score: available in 308 patients;

^d^ Lab values >10% missings in: neutrophil count, albumin;

^e^ CP-score (assumed no ascites and hepatic encephalopathy at start of treatment): available in 223 patients

### Registration trials and outcomes eligible vs. ineligible

**Fig 2:**
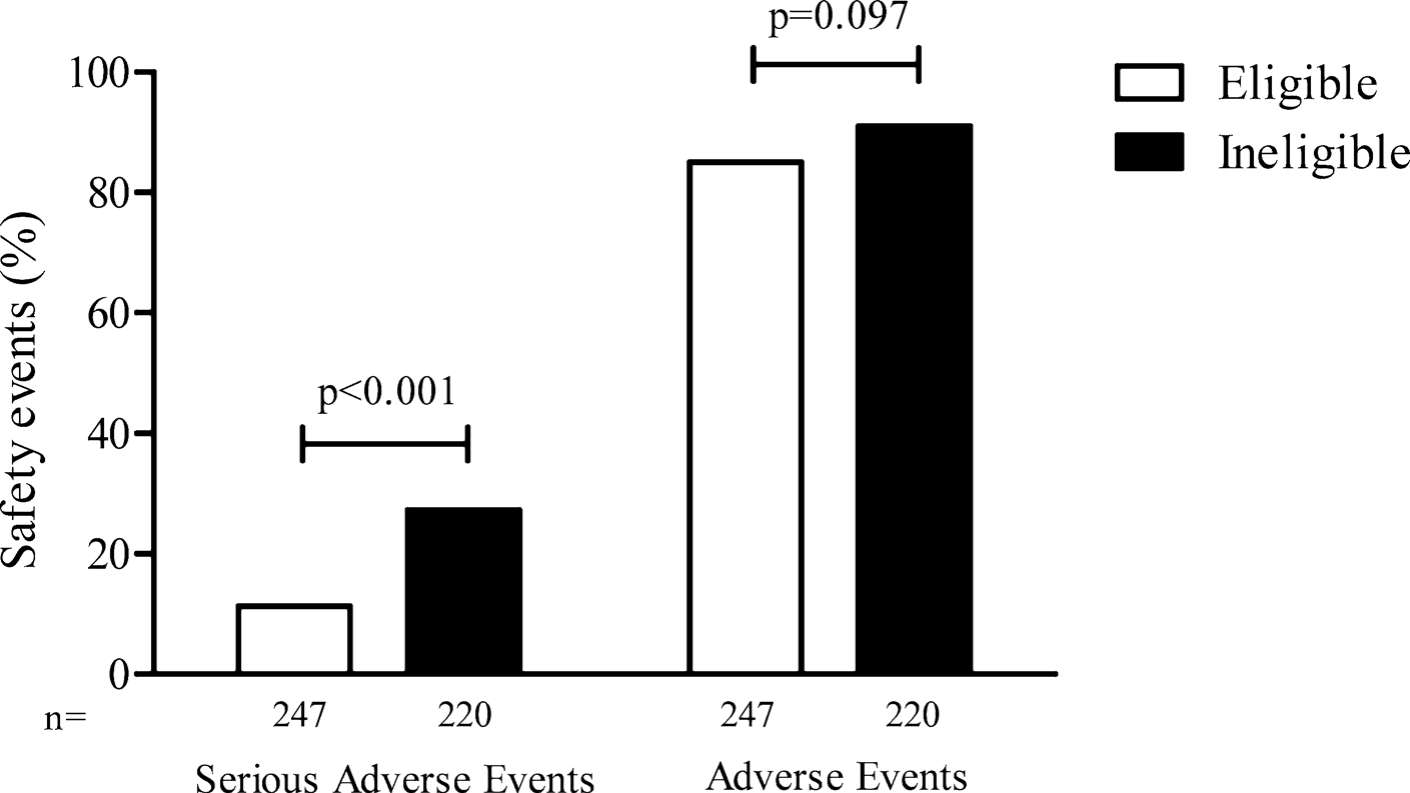
Safety in real world patients who would be eligible and ineligible for registration trials. The bars represent the proportion of patients who experienced a serious adverse event or adverse event in patients eligible or ineligible for registration trials.

**Fig 3:**
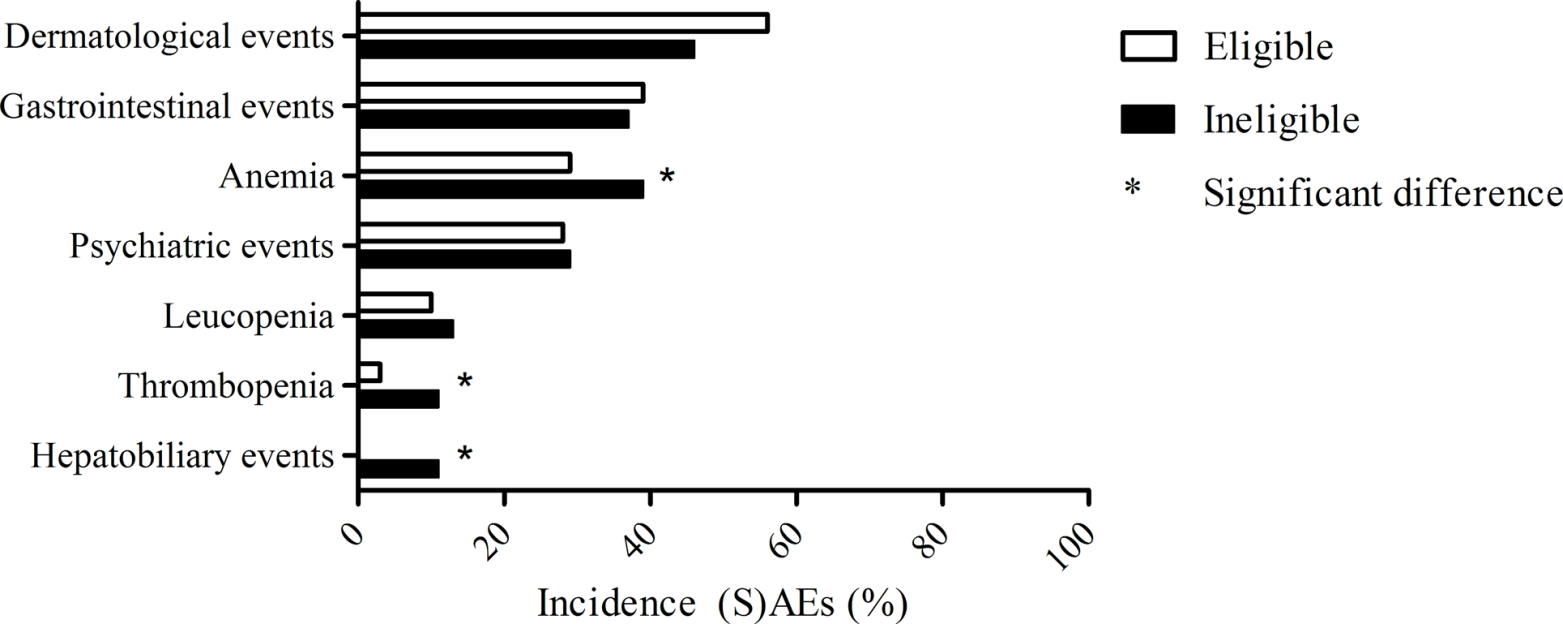
Incidence of specific (serious) adverse events in eligible and ineligible patients. The bars represent the incidence of various categories of (serious) adverse events between patients eligible and ineligible for registration trials. The asterix (*) marks significant differences between eligible and ineligible patients.

Our search yielded eight trials of telaprevir and boceprevir [17–24], and five registration trials were included. (S1 Table). [22–24] On the basis of the general criteria (Table 1), 47% of patients treated in real world practice would be excluded from registration trials. We than compared the eligible to ineligible population with respect to safety parameters. We found that ineligible patients had significantly more SAEs compared to eligible patients (27% vs. 11%, p<0.001) (Fig 2). A total of 37 SAEs occurred in 28 eligible patients (1 patient died due to an accident), compared to 103 SAEs which occurred in 60 ineligible patients (7 patients died) (S5 Table). Also, after excluding patients with a history of decompensated liver disease (n = 24) from the analysis, ineligible patients had significantly higher SAE rates (24% vs. 11%, p<0.001). Further, ineligible patients had a higher median number of AEs and SAEs (p = 0.039 and p<0.001 respectively, S6 Table). The incidence of some typical hepatic or therapy related (S)AEs (anemia, thrombopenia and hepatobiliary events) were significantly higher in the ineligible patients (Fig 3).

**Fig 4:**
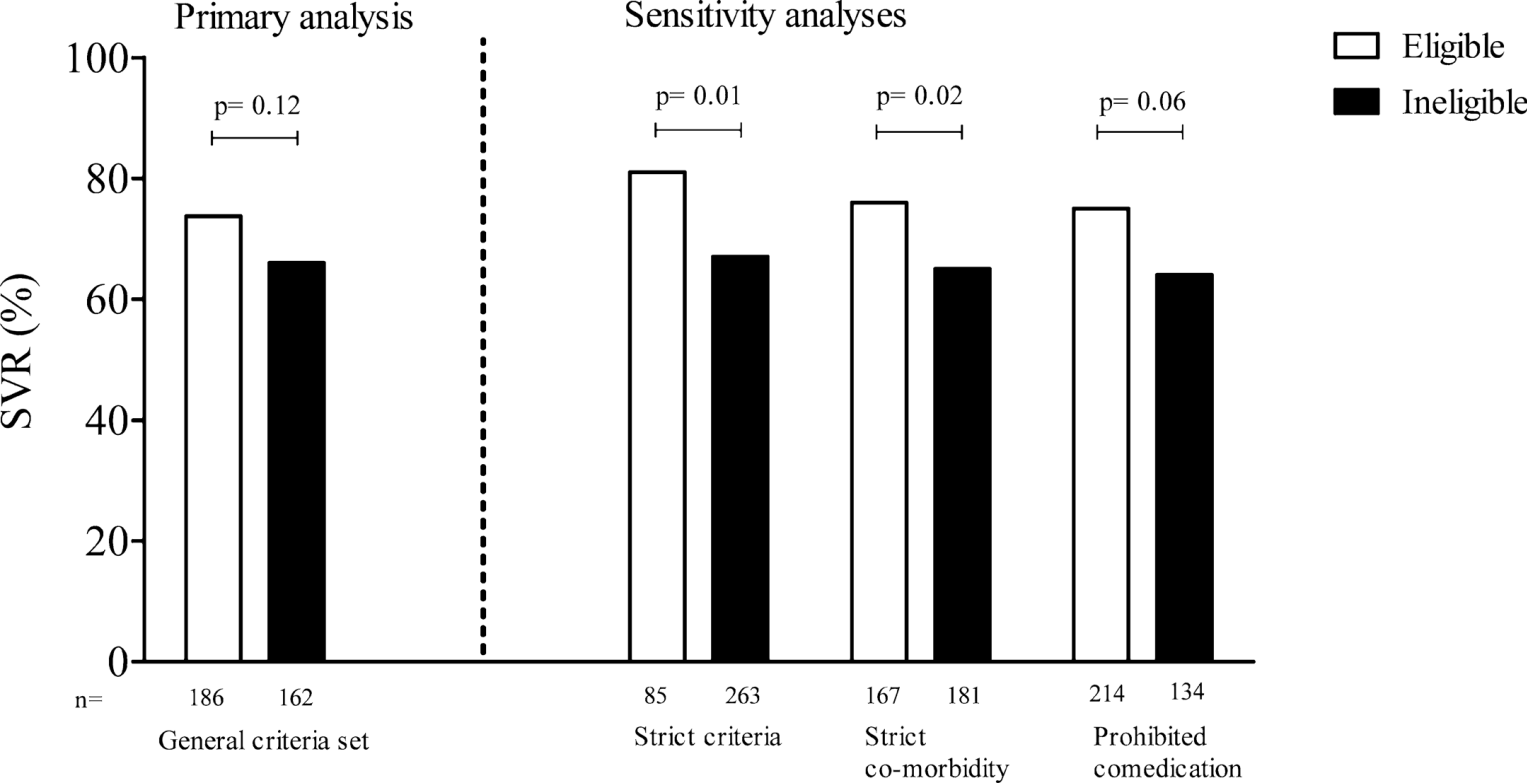
Effectiveness in real world treatment naive and relapse patients who would be eligible and ineligible for registration trials. Primary and sensitivity analyses on effectiveness of therapy in eligible vs. ineligible naive and relapse patients (n = 348). The bars represent the proportion of patients who reached a sustained virological response (SVR) within the groups. For sensitivity analyses different criteria sets are used to determine eligibility of patients, hence different numbers of patients in both groups.

We found (non-significant) lower SVR rates in ineligible patients. Two sensitivity analyses detected lower SVR rates in ineligible patients (treatment naive–relapse group): when applying most strict criteria (81% vs. 67%, p = 0.01) or when most stringent exclusion of patients with co-morbidity was done (76% vs. 65%, p = 0.02). We observed no difference in SVR in the third sensitivity analysis, where we excluded concomitant medication from the criteria set (Fig 4). No significant differences in effectiveness were found in the non-responder group (S1 Fig).

### Criteria for ineligibility

Most important criteria for ineligibility were related to co-morbidity and signs or history of hepatic decompensation. In 220 ineligible patients, main reason for exclusion was the use of prohibited concomitant medication (n = 65), followed by anemia (n = 25), psychiatric co-morbidity (n = 24), and current or history of decompensated liver disease (n = 24). Median number of exclusion criteria within a patient was 1 (range 1–6). Univariable analysis showed most important criteria associated with lack of SVR, i.e. current or history of decompensated liver disease (RR 0.66), platelet count (RR 0.58), albumin (RR 0.49), bilirubin (RR 0.58) and neutrophil count (RR 0.55). Similar criteria were associated with a higher risk on an SAE: a history of decompensated liver disease (RR 1.81), platelet count (RR 1.45), albumin (RR 2.03), bilirubin (RR1.89), hemoglobin (RR 1.72), malignancy (RR 2.31) and presence of cardiac disease (RR 1.97). Outcomes of these analyses are depicted in Table 3.

**Table 3:** Top criteria which impact trial eligibility.

Criterion	n	% of ineligible patients	RR on SVR(95% CI)	RR on SAE(95% CI)
Prohibited comedication listed in protocols	65	30	0.99 (0.70–1.39)	1.17 (1.00–1.38)
Hemoglobin <12 g/dL (females) or <13 g/dL (males)	25	11	0.69 (0.46–1.02)	**1.72 (1.14–2.60)**
Presence of severe psychiatric disease	24	11	1.27 (0.67–2.40)	1.03 (0.84–1.72)
Current or history of ascites, encephalopathy or bleeding varices	24	11	**0.66 (0.44–0.97)**	**1.81 (1.17–2.81)**
Platelet count < 90 x10^9^/L	23	11	**0.58 (0.41–0.82)**	**1.45 (1.01–2.08)**
Presence of hemophilia	23	11	1.42 (0.71–2.85)	4.51 (0.66–30.93)*
Serum albumin < 3.3 g/dL	22	10	**0.49 (0.36–0.68)**	**2.03 (1.23–3.37)**
Total bilirubin > 1,8x ULN†	16	7	**0.58 (0.39–0.86)**	**1.89 (1.08–3.29)**
TSH > 1.2 x ULN or 0.8x LLN†	14	6	0.71 (0.41–1.22)	1.34 (0.36–4.90)*
Active malignant disease or malignant disease in past 5 years (except basal cell carcinoma)	14	6	1.02 (0.50–2.09)	**2.31 (1.14–4.66)**
Central nervous system disorder present	13	6	0.78 (0.43–1.43)	1.18 (0.82–1.70)
Significant cardiac disease present	12	6	1.46 (0.54–3.91)	**1.97 (1.01–3.86)**
Presence of auto-immune disease	11	5	1.34 (0.51–3.55)	1.50 (0.87–2.58)
Absolute neutrophil count < 1.2 x10^9^/L	9	4	**0.55 (0.34–0.90)**	1.62 (0.25–10.43)*
Ultrasound with no signs of HCC	6	3	0.74 (0.33–1.66)	1.64 (0.74–3.65)
Creatinine clearance ≤ 50 ml/min	5	2	0.63 (0.30–1.30)	2.05 (0.70–6.01)
AST 10 x ULN†	5	2	0.61 (0.29–1.26)	1.01 (0.65–1.57)
Presence of another liver disease	5	2	0.60 (0.29–1.24)	-

* Poisson regression when log binomial regression did not converge

† ULN = upper limit of normal; LLN = lower limit of normal

## Discussion

This study sheds doubt on the generalizability of registration trials to the real world CHC population. In our study, one of the key findings is that nearly half of treated CHC patients would be ineligible for registration trials. Most important exclusion criteria relate to signs or history of hepatic decompensation and co-morbidity (cardiac disease, anemia, malignancy and neutropenia). Patients meeting those exclusion criteria developed more SAEs (RR between 1.45 and 2.31) and were less likely to reach SVR (RR between 0.49 and 0.66), especially when strict criteria were used. Vice versa, eligible patients had SVR and SAE rates comparable to published trials.[17–21] Altogether, this indicates that results from registration trials are only generalizable to the real world patients who fulfill the eligibility criteria. Translating results originating from registration trials to patients that would be ineligible should be done with caution.

The difference between registration trials and real world reflects a ‘development paradox’. Drugs are developed through a phase II-III program that targets easy-to-treat patients, while in the real world difficult-to-treat patients are prioritized for treatment.[1, 25, 26] The sequence of drug development starting with easy-to-treat patients seems appropriate, but the final hurdle to perform trials that specifically target difficult-to-treat patients is often sidestepped or delayed until after market authorization. As a result, this population who has a clear treatment indication is exposed to DAAs in the real world, without proper data on efficacy and toxicity.[27] This results in an increased proportion of adverse events, dropouts and hence lower effectiveness.[28] Our results support the ‘development paradox’ and provide reasons why real world outcomes do differ from registration trials.

Our data on limited generalizability of registration trials accords with the literature. An increased likelihood for SAEs in patients with a history of decompensated cirrhosis who would have been excluded from registration trials was reported in a large CHC cohort (n = 2084). [9] Some 30–47% of compensated cirrhotic patients treated with first-generation protease inhibitors would be ineligible for registration trials, and this study showed unexpected high SAE rates in that population.[7] In addition, a study on ledipasvir/sofosbuvir in advanced liver disease patients, published after FDA and EMA approval, reported much higher SAE rates (23%) compared to registration trials (3%). [29] For another CHC regimen, paritaprevir/ritonavir, ombitasvir and dasabuvir, the FDA label changed within one year following approval based on review of adverse events. This regime is now contra-indicated in patients with Child-Pugh B cirrhosis. [30] It is likely that this could have been prevented if these patients had been trialed prior to approval of the regimen. There is literature that suggests that serious adverse events might be related to disease course instead of therapy.[31] Nonetheless, timely controlled studies in CHC patients with decompensated liver disease are necessary to accurately gauge risk-benefit balance for these individual patients.

Here, we used the first-generation protease inhibitor treated patients as an example cohort. We believe that our results are also applicable to new generation DAAs, because eligibility criteria of registration trials are comparable to the set used in the current study (S7 Table). [31–37] Indeed, a Canadian HIV/HCV cohort, found that up to 94% of patients from that cohort would be ineligible for registration trials with new generation DAAs.[10] Furthermore a real world cohort showed that liver decompensation and SAEs during sofosbuvir containing regimens were associated with lower baseline albumin and higher total bilirubin, which are general exclusion criteria. [38] As toxicity of new generation DAAs decreases, the difference between trials and real world might become smaller, however with the high ineligibility rate of real world patients, generalization of results remains difficult.

Limited generalizability of registration trials is also seen in other liver diseases such as hepatocellular carcinoma (HCC) and HBV infection. For example, sorafenib was approved for HCC treatment, on the basis of studies that excluded Child-Pugh B and C cirrhotic patients. [39, 40] A real world cohort reported significantly decreased overall survival with sorafenib in Child-Pugh B compared to Child-Pugh A cirrhotics.[41] Likewise, post-marketing studies in entecavir for chronic HBV infection show lower proportions of ALT normalization than was shown in registration trials. [42]

Our study comes with strengths and limitations. Strengths of this study are the nationwide and multicenter character, resulting in a large and representative real world cohort. Limitations of this study are the retrospective character that resulted in (some) missing values. We handled this conservatively, by classifying the missing value as eligible for that criterion. Furthermore, chart review may result in reporting bias, but we used strict definitions to reduce this. Another limitation is that patients received first-generation protease inhibitors, peginterferon and ribavirin, which may increase the potential for toxicity. However, we think that our results are also valid for new generation DAAs.

In conclusion, nearly half of CHC patients treated in real world practice would be ineligible for registration trials. In these patients we found impaired safety and effectiveness related to specific eligibility criteria (hepatic decompensation, co-morbidity). Prior to regulatory approval, new drugs should also be studied in the difficult-to-treat population, including patients with hepatic decompensation and co-morbidity, to genuinely assess the benefits and risks of treatment in the real world population.

## Supporting Information

S1 Fig Effectiveness in real world treatment nonresponder and other patients who would be eligible and ineligible for registration trials. Primary and sensitivity analyses on effectiveness of therapy in eligible vs. ineligible nonresponder or other patients (n = 118). For sensitivity analyses different criteria sets are used to determine eligibility of patients, hence different numbers of patients in groups.
(DOCX)

S1 Table Search strategy. This is the flowchart of the systematic search for registration trials with telaprevir and boceprevir.
(DOCX)

S2 Table Set of least and most stringent combined inclusion and exclusion criteria of registration trials. This table shows the least stringent and most stringent criteria of different registration trials per variable. The least stringent criteria set was used for primary analyses and the most stringent criteria set for a sensitivity analysis.
(DOCX)

S3 Table Baseline characteristics telaprevir and boceprevir treated patients. Table including baseline characteristics of patients treated with telaprevir and boceprevir, these patients are pooled in the primary analysis.
(DOCX)

S4 Table Effectiveness and safety of telaprevir compared to boceprevir. Table showing effectiveness and safety results of telaprevir vs. boceprevir, these patients are pooled in the primary analysis.
(DOCX)

S5 Table Serious adverse events categories. Table with categories of serious adverse events in eligible vs. ineligible patients.
(DOCX)

S6 Table Sensitivity analyses. Table showing outcomes of sensitivity analyses: analysis with most stringent criteria, analysis with strict exclusion of patients with co-morbidity, analysis without prohibited comedication as exclusion criterion.
(DOCX)

S7 Table Exclusion criteria of registration trials in new generation DAAs. Table with exclusion criteria of new generation DAAs in comparison to our general criteria set.
(DOCX)

## Abbreviations

AE: Adverse Event
CHC: Chronic Hepatitis C
CNS: Central Nervous System
CTCAE: Common Terminology Criteria for Adverse Events
DAA: Direct-Acting Antiviral
EMA: European Medicines Agency
FDA: Food and Drug Administration
HBV: Hepatitis B Virus
HCC: Hepatocellular Carcinoma
HCV: Hepatitis C Virus
HIV: Human Immunodeficiency Virus
INR: International Normalized Ratio
RNA: RiboNucleic Acid
RR: Relative Risk
SAE: Serious Adverse Event
SVR: Sustained Virological Response
TSH: Thyroid Stimulating Hormone

## References

[1] European Association for Study of L. EASL Recommendations on Treatment of Hepatitis C 2015. Journal of hepatology. 2015;63(1):199–236. 10.1016/j.jhep.2015.03.025 . 25911336

[2] Panel AIHG. Hepatitis C guidance: AASLD-IDSA recommendations for testing, managing, and treating adults infected with hepatitis C virus. Hepatology. 2015;62(3):932–54. 10.1002/hep.27950 . 26111063

[3] Peppercorn JM, Weeks JC, Cook EF, Joffe S. Comparison of outcomes in cancer patients treated within and outside clinical trials: conceptual framework and structured review. Lancet. 2004;363(9405):263–70. 10.1016/S0140-6736(03)15383-4 . 14751698

[4] Kievit W, Fransen J, Oerlemans AJ, Kuper HH, van der Laar MA, de Rooij DJ, et al The efficacy of anti-TNF in rheumatoid arthritis, a comparison between randomised controlled trials and clinical practice. Annals of the rheumatic diseases. 2007;66(11):1473–8. 10.1136/ard.2007.072447 17426065 PMC2111629

[5] Ha C, Ullman TA, Siegel CA, Kornbluth A. Patients enrolled in randomized controlled trials do not represent the inflammatory bowel disease patient population. Clinical gastroenterology and hepatology: the official clinical practice journal of the American Gastroenterological Association. 2012;10(9):1002–7; quiz e78. 10.1016/j.cgh.2012.02.004 . 22343692

[6] Muir AJ. The rapid evolution of treatment strategies for hepatitis C. The American journal of gastroenterology. 2014;109(5):628–35; quiz 36. 10.1038/ajg.2014.66 . 24732866

[7] Hezode C, Fontaine H, Dorival C, Zoulim F, Larrey D, Canva V, et al Effectiveness of telaprevir or boceprevir in treatment-experienced patients with HCV genotype 1 infection and cirrhosis. Gastroenterology. 2014;147(1):132–42 e4. 10.1053/j.gastro.2014.03.051 . 24704719

[8] Sulkowski MS, Vargas HE, Di Bisceglie AM, Kuo PA, Reddy KR, Lim JK, et al Effectiveness of Simeprevir plus Sofosbuvir, With or Without Ribavirin, in Real-World Patients with HCV Genotype 1 Infection. Gastroenterology. 2015 10.1053/j.gastro.2015.10.013 . 26497081 PMC4727992

[9] Gordon SC, Muir AJ, Lim JK, Pearlman B, Argo CK, Ramani A, et al Safety profile of boceprevir and telaprevir in chronic hepatitis C: real world experience from HCV-TARGET. Journal of hepatology. 2015;62(2):286–93. 10.1016/j.jhep.2014.08.052 25218788 PMC4586075

[10] Saeed S, Strumpf EC, Walmsley S, Rollet-Kurhajec K, Pick N, Martel-Laferriere V, et al How generalizable are the results from trials of Direct Antiviral Agents to people coinfected with HIV/Hepatitis C virus in the real world? Clinical infectious diseases: an official publication of the Infectious Diseases Society of America. 2016 10.1093/cid/civ1222 . 26743093 PMC4787608

[11] Lamers MH, Broekman MM, Boucher CA, Brouwer JT, Burger DM, van Hoek B, et al Treatment of hepatitis C monoinfection in adults—Dutch national guidelines. The Netherlands journal of medicine. 2013;71(7):377–85. . 24038567

[12] HHS. U, FDA., CDER., CBER. Guidance for Industry and Investigators. Available: http://wwwfdagov/downloads/Drugs//Guidances/UCM227351pdf. 2012.

[13] National Cancer Institute N, NIH, DHHS. Common Terminology Criteria for Adverse Events v4.0 2009.

[14] Kieran J, Schmitz S, O'Leary A, Walsh C, Bergin C, Norris S, et al The relative efficacy of boceprevir and telaprevir in the treatment of hepatitis C virus genotype 1. Clinical infectious diseases: an official publication of the Infectious Diseases Society of America. 2013;56(2):228–35. 10.1093/cid/cis880 . 23074309

[15] AASLD, IDSA. HCV Guidance: Recommendations for Testing, Managing, and Treating Hepatitis C. 2016.

[16] Zhang J, Yu KF. What's the relative risk? A method of correcting the odds ratio in cohort studies of common outcomes. Jama. 1998;280(19):1690–1. . 9832001 10.1001/jama.280.19.1690

[17] Zeuzem S, Andreone P, Pol S, Lawitz E, Diago M, Roberts S, et al Telaprevir for retreatment of HCV infection. The New England journal of medicine. 2011;364(25):2417–28. 10.1056/NEJMoa1013086 . 21696308

[18] Jacobson IM, McHutchison JG, Dusheiko G, Di Bisceglie AM, Reddy KR, Bzowej NH, et al Telaprevir for previously untreated chronic hepatitis C virus infection. The New England journal of medicine. 2011;364(25):2405–16. 10.1056/NEJMoa1012912 . 21696307

[19] Sherman KE, Flamm SL, Afdhal NH, Nelson DR, Sulkowski MS, Everson GT, et al Response-guided telaprevir combination treatment for hepatitis C virus infection. The New England journal of medicine. 2011;365(11):1014–24. 10.1056/NEJMoa1014463 21916639 PMC3809077

[20] Bacon BR, Gordon SC, Lawitz E, Marcellin P, Vierling JM, Zeuzem S, et al Boceprevir for previously treated chronic HCV genotype 1 infection. The New England journal of medicine. 2011;364(13):1207–17. 10.1056/NEJMoa1009482 21449784 PMC3153125

[21] Poordad F, McCone J Jr., Bacon BR, Bruno S, Manns MP, Sulkowski MS, et al Boceprevir for untreated chronic HCV genotype 1 infection. The New England journal of medicine. 2011;364(13):1195–206. 10.1056/NEJMoa1010494 21449783 PMC3766849

[22] Buti M, Agarwal K, Horsmans Y, Sievert W, Janczewska E, Zeuzem S, et al Telaprevir twice daily is noninferior to telaprevir every 8 hours for patients with chronic hepatitis C. Gastroenterology. 2014;146(3):744–53 e3. 10.1053/j.gastro.2013.11.047 . 24316262

[23] Flamm SL, Lawitz E, Jacobson I, Bourliere M, Hezode C, Vierling JM, et al Boceprevir with peginterferon alfa-2a-ribavirin is effective for previously treated chronic hepatitis C genotype 1 infection. Clinical gastroenterology and hepatology: the official clinical practice journal of the American Gastroenterological Association. 2013;11(1):81–7 e4; quiz e5. 10.1016/j.cgh.2012.10.006 . 23064222

[24] Vierling JM, Davis M, Flamm S, Gordon SC, Lawitz E, Yoshida EM, et al Boceprevir for chronic HCV genotype 1 infection in patients with prior treatment failure to peginterferon/ribavirin, including prior null response. Journal of hepatology. 2014;60(4):748–56. 10.1016/j.jhep.2013.12.013 . 24362076

[25] Barua S, Greenwald R, Grebely J, Dore GJ, Swan T, Taylor LE. Restrictions for Medicaid Reimbursement of Sofosbuvir for the Treatment of Hepatitis C Virus Infection in the United States. Annals of internal medicine. 2015;163(3):215–23. 10.7326/M15-0406 . 26120969

[26] Ferenci P, Dusheiko G. Beyond phase 3 registration trials: defining safety for triple therapy with protease inhibitors in cirrhosis. Gut. 2014;63(7):1033–4. 10.1136/gutjnl-2013-306480 . 24334256

[27] van der Meer AJ, Veldt BJ, Feld JJ, Wedemeyer H, Dufour JF, Lammert F, et al Association between sustained virological response and all-cause mortality among patients with chronic hepatitis C and advanced hepatic fibrosis. Jama. 2012;308(24):2584–93. 10.1001/jama.2012.144878 . 23268517

[28] Majid A, McAninch J, Morgan DJ, El Kamary SS, Zhan M, Kapelusznik L, et al Predictors of early treatment discontinuation in a cohort of patients treated with boceprevir-based therapy for hepatitis C infection. Journal of viral hepatitis. 2014;21(8):585–9. 10.1111/jvh.12201 . 24224781

[29] Charlton M, Everson GT, Flamm SL, Kumar P, Landis C, Brown RS Jr., et al Ledipasvir and Sofosbuvir Plus Ribavirin for Treatment of HCV Infection in Patients With Advanced Liver Disease. Gastroenterology. 2015;149(3):649–59. 10.1053/j.gastro.2015.05.010 . 25985734

[30] FDA. FDA Drug Safety Communication: FDA warns of serious liver injury risk with hepatitis C treatments Viekira Pak and Technivie. 2015.

[31] Saxena V, Nyberg L, Pauly M, Dasgupta A, Nyberg A, Piasecki B, et al Safety and Efficacy of Simeprevir/Sofosbuvir in Hepatitis C-Infected Patients With Compensated and Decompensated Cirrhosis. Hepatology. 2015;62(3):715–25. 10.1002/hep.27922 26033798 PMC4549204

[32] Alqahtani SA, Afdhal N, Zeuzem S, Gordon SC, Mangia A, Kwo P, et al Safety and tolerability of ledipasvir/sofosbuvir with and without ribavirin in patients with chronic hepatitis C virus genotype 1 infection: Analysis of phase III ION trials. Hepatology. 2015;62(1):25–30. 10.1002/hep.27890 . 25963890

[33] Feld JJ, Kowdley KV, Coakley E, Sigal S, Nelson DR, Crawford D, et al Treatment of HCV with ABT-450/r-ombitasvir and dasabuvir with ribavirin. The New England journal of medicine. 2014;370(17):1594–603. 10.1056/NEJMoa1315722 . 24720703

[34] Poordad F, Hezode C, Trinh R, Kowdley KV, Zeuzem S, Agarwal K, et al ABT-450/r-ombitasvir and dasabuvir with ribavirin for hepatitis C with cirrhosis. The New England journal of medicine. 2014;370(21):1973–82. 10.1056/NEJMoa1402869 . 24725237

[35] Zeuzem S, Jacobson IM, Baykal T, Marinho RT, Poordad F, Bourliere M, et al Retreatment of HCV with ABT-450/r-ombitasvir and dasabuvir with ribavirin. The New England journal of medicine. 2014;370(17):1604–14. 10.1056/NEJMoa1401561 . 24720679

[36] Andreone P, Colombo MG, Enejosa JV, Koksal I, Ferenci P, Maieron A, et al ABT-450, ritonavir, ombitasvir, and dasabuvir achieves 97% and 100% sustained virologic response with or without ribavirin in treatment-experienced patients with HCV genotype 1b infection. Gastroenterology. 2014;147(2):359–65 e1. 10.1053/j.gastro.2014.04.045 . 24818763

[37] Ferenci P, Bernstein D, Lalezari J, Cohen D, Luo Y, Cooper C, et al ABT-450/r-ombitasvir and dasabuvir with or without ribavirin for HCV. The New England journal of medicine. 2014;370(21):1983–92. 10.1056/NEJMoa1402338 . 24795200

[38] Perumalswami PV, Patel N, Bichoupan K, Ku L, Yalamanchili R, Harty A, et al High baseline bilirubin and low albumin predict liver decompensation and serious adverse events in HCV-infected patients treated with sofosbuvir-containing regimens. Journal of viral hepatitis. 2016 10.1111/jvh.12530 . 26989855

[39] FDA. Full Prescribing Information Nexavar. 2013;(Reference ID: 3411803).

[40] Llovet JM, Ricci S, Mazzaferro V, Hilgard P, Gane E, Blanc JF, et al Sorafenib in advanced hepatocellular carcinoma. The New England journal of medicine. 2008;359(4):378–90. 10.1056/NEJMoa0708857 . 18650514

[41] Kim HY, Park JW, Joo J, Kim H, Woo SM, Lee WJ, et al Worse outcome of sorafenib therapy associated with ascites and Child-Pugh score in advanced hepatocellular carcinoma. Journal of gastroenterology and hepatology. 2013;28(11):1756–61. 10.1111/jgh.12310 . 23800278

[42] Ahn J, Lee HM, Lim JK, Pan CQ, Nguyen MH, Ray Kim W, et al Entecavir safety and effectiveness in a national cohort of treatment-naive chronic hepatitis B patients in the US—the ENUMERATE study. Alimentary pharmacology & therapeutics. 2016;43(1):134–44. 10.1111/apt.13440 . 26510638 PMC4926997

