# Supplementary material for: Limited Generalizability of Registration Trials in Hepatitis C: A Nationwide Cohort Study
Source: PLoS One. 2016 Sep 6;11(9):e0161821. doi: 10.1371/journal.pone.0161821 (PMC5012685; doi:10.1371/journal.pone.0161821)
Supplement: S1 Fig — Primary and sensitivity analyses on effectiveness of therapy in eligible vs. ineligible nonresponder or other patients (n = 118). For sensitivity analyses different criteria sets are used to determine eligibility of patients, hence different numbers of patients in groups. (DOCX) [file pone.0161821.s001.docx]

**S1 Fig. Effectiveness in real world nonresponder and other patients who would be eligible and ineligible for registration trials**
