# Supplementary material for: Limited Generalizability of Registration Trials in Hepatitis C: A Nationwide Cohort Study
Source: PLoS One. 2016 Sep 6;11(9):e0161821. doi: 10.1371/journal.pone.0161821 (PMC5012685; doi:10.1371/journal.pone.0161821)
Supplement: S1 Table — This is the flowchart of the systematic search for registration trials with telaprevir and boceprevir. (DOCX) [file pone.0161821.s002.docx]

**S1 Table. Search strategy**

FDA Telaprevir label (Reference ID: 3397093)

Clinical studies

- Treatment naïve adults: trial 108 (ADVANCE), trial 111 (ILLUMINATE), trial C211 (OPTIMIZE)
- Treatment experienced adults: trial C216 (REALIZE)

FDA Boceprevir label (Revised 05/2011)

Clinical studies

- Treatment naïve adults: SPRINT-2
- Treatment experienced adults: RESPOND-2

Clinical trials.gov

Search #2 Boceprevir: 54 studies

Additional criteria: phase 3:17 hits

Search #1 Telaprevir OR VX 950: 93 studies

Additional criteria: phase 3: 26 hits

Exclusion of studies

- No phase 3:
- HIV/coinfection: 2
- Another regime: 2
- Subgroups: 5 (menopausal women, pediatric, EPO vs dose reduction, IL28B CC, eltrombopag
- Asia: 2
- Russia: 1
- Other genotype: 2

Exclusion of studies

- Roll over: 1
- HIV/coinfection: 3
- HCC: 1
- Post-livertransplantation: 1
- Another regime: 8
- Other peginterferon: 5
- Subgroups: 1 (IL28B CC)
- Russian: 1
- Early access: 1

3 trials selected

- NCT00705432 - SPRINT-2
- NCT00708500 - RESPOND-2
- NCT00845065

4 trials selected

- NCT01241760 - OPTIMIZE
- NCT00703118 - REALIZE
- NCT00627926 - ADVANCE
- NCT00758043 - ILLUMINATE

Pubmed database

| **No** | **Search** | **Results** | **Exclusion** |
| --- | --- | --- | --- |
| **#1** | Telaprevir AND phase 3  *Limits:*   - *clinical trial* - *date: 1-1-2000 until 1-1-2014* | 31 results:  Inclusion:   - NCT01241760 - OPTIMIZE - NCT00703118 - REALIZE - NCT00627926 - ADVANCE | Exclusion on title and abstract: 27  Exclusion on full text: 1 (Japan) |
| **#2** | Boceprevir AND phase 3  *Limits:*   - *clinical trial* - *date: 1-1-2000 until 1-1-2014* | 25 results  Inclusion   - NCT00910624 - PROVIDE - NCT00845065 - NCT00705432 - SPRINT-2 - NCT00708500 - RESPOND-2 | Exclusion on title and abstract: 21 |

Conclusion search in FDA label, clinical trials.gov and pubmed:

|  | **Telaprevir** | **Boceprevir** |
| --- | --- | --- |
| **Inclusion** | - NCT00703118 - REALIZE - NCT00627926 - ADVANCE - NCT00758043 - ILLUMINATE | - NCT00705432 - SPRINT-2 - NCT00708500 - RESPOND-2 |
| **Exclusion** | - NCT01241760 – OPTIMIZE: no full eligibility criteria available | - NCT00910624 – PROVIDE: roll over of SPRINT-1, SPRINT-2 and RESPOND-2, so identical inclusion criteria - NCT00845065 (identical inclusion criteria RESPOND-2) |
