# Supplementary material for: Limited Generalizability of Registration Trials in Hepatitis C: A Nationwide Cohort Study
Source: PLoS One. 2016 Sep 6;11(9):e0161821. doi: 10.1371/journal.pone.0161821 (PMC5012685; doi:10.1371/journal.pone.0161821)
Supplement: S2 Table — This table shows the least stringent and most stringent criteria of different registration trials per variable. The least stringent criteria set was used for primary analyses and the most stringent criteria set for a sensitivity analysis. (DOCX) [file pone.0161821.s003.docx]

**S2 Table. Set of least and most stringent combined inclusion and exclusion criteria of registration trials**

| **Variable** | **Least stringent criteria** | **Most stringent criteria** |
| --- | --- | --- |
| **Inclusion** |  |  |
| Age | Subject ≥ 18 years | Subject 18-70 years |
| HCV RNA | HCV RNA detectable | HCV RNA ≥ 10.000 |
| Weight | Between 40-125 kg | Between 40-125 kg |
| Hepatocellular carcinoma (HCC) | Ultrasound with no signs of HCC | Ultrasound with no signs of HCC |
| **Exclusion** |  |  |
| Genotype | HCV with > 1 subtype or genotype | HCV with > 1 subtype or genotype |
| Treatment history | Ignore this criterion | Exclusion of nullresponders, viral breakthrough and early discontinuation |
| Hemoglobin (Hb) | Hb <12 g/dL for females or <13 g/dL for males | Hb <12 g/dL for females or <13 g/dL for males |
| Neutrophil count | Absolute neutrophil count <1.2 x10^9^/L | Absolute neutrophil count <1.5 x10^9^/L |
| Platelet count | Platelet count <90 x10^9^/L | Platelet count <100 x10^9^/L |
| Renal insufficiency | Creatinine clearance ≤ 50 ml/min | Creatinine > ULN† |
| Albumin | Serum albumin < 3.3 g/dL | Serum albumin < LLN† |
| Bilirubin | total bilirubin > 1,8x ULN† | total bilirubin > 1,6x ULN † |
| Glucose | Ignore this criterion | Serum glucose ≥ 140 mg/dL (nonDM) |
| Protrombin Time (PT)/INR | INR ≥ 1.5 | PT > 10% ULN† |
| TSH | TSH > 1.2 x ULN or 0.8x LLN† | TSH above or below normal range |
| ALT | ALT 10 x ULN† | ALT 10 x ULN† |
| AST | AST 10 x ULN† | AST 10 x ULN† |
| Contra-indication to peginterferon/ribavirin   - Hemoglobinopathy - Cardiac disease^1^ - Renal insufficiency | Hemoglobinopathy present  Significant cardiac disease present  See renal insufficiency | Hemoglobinopathy present ǂ  Significant cardiac disease present ǂ  See renal insufficiency ǂ |
| Auto-immune disease^2^ | Presence of auto-immune disease | Presence of auto-immune disease ǂ |
| COPD | COPD gold III or IV | COPD gold I-IV and unknown gold |
| Current or history of decompensated liver disease | History of ascites, encephalopathy or bleeding varices | History of ascites, encephalopathy or bleeding varices ǂ |
| Other liver disease | Other liver disease | Other liver disease ǂ |
| Malignancy | Active malignant or malignant disease in past 5 years (except basal cell carcinoma) | Active malignant or malignant disease in past 5 years (except basal cell carcinoma) ǂ |
| Pancreatitis | Acute pancreatitis in past 5 years | History of acute or chronic pancreatitis |
| Retinopathy | Retinopathy present | Retinopathy present ǂ |
| Seizure | Seizure disorder requiring medication | History of seizure disorder |
| Transplantation | Patient with a history of an organ transplant | Patient with a history of an organ transplant ǂ |
| Psychiatric comorbidity^3^ | Severe psychiatric disease | Moderate and severe psychiatric disease |
| Corticosteroids use | Use of systemic corticosteroids | Use of systemic corticosteroids ǂ |
| Alcohol or drugs use | Ignore this criterion | Alcohol use > 2 IE/day or drugs use |
| Hemophilia | Hemophilia | Hemophilia ǂ |
| Central Nervous System disorder/Stroke/TIA^4^ | CNS disorder present | CNS disorder present ǂ |
| Malabsorption | History of malabsorption disorder | History of malabsorption disorder ǂ |
| Indwelling cathether | Subject with indwelling venous catheter | Subject with indwelling venous catheter ǂ |
| **Comedication** | Comedication literally on prohibited medication list of protocol | Comedication in the same anatomical therapeutic code (ATC)-group as prohibited medication list of protocol |

^1^ Significant cardiac disease was defined as: current or history of unstable cardiac disease (angina, congestive heart failure, recent myocardial infarction, pulmonary hypertension, complex congenital heart disease, cardiomyopathy, and/or significant arrhythmia)
^2^Auto-immune disease was defined as: immunologically mediated disease (inflammatory bowel disease, celiac disease, rheumatoid arthritis, idiopathic thrombocytopenic purpura, systemic lupus erythematosus, autoimmune hemolytic anemia, scleroderma, sarcoidosis, severe psoriasis, or autoimmune hepatitis)
^3^ Psychiatric comorbidity was defined as: severe depression or hospitalization for depression, schizophrenia, bipolar illness, severe anxiety or personality disorder, a period of disability or impairment due to a psychiatric disease within the past 5 years
^4^ CNS disorder was defined as: CNS trauma requiring intubation, intracranial pressure monitoring, brain meningeal/skull surgery, or resulting in seizure, coma, neurologic deficits, abnormal brain imaging, CSF leak, prior brain hemorrhage and/or intracranial aneurysms, or history of stroke or transient ischemic attack (TIA)
† ULN = upper limit of normal; LLN = lower limit of normal
ǂ plus exclusion of cases with insufficient information about specific co-morbidity
