# Supplementary material for: Limited Generalizability of Registration Trials in Hepatitis C: A Nationwide Cohort Study
Source: PLoS One. 2016 Sep 6;11(9):e0161821. doi: 10.1371/journal.pone.0161821 (PMC5012685; doi:10.1371/journal.pone.0161821)
Supplement: S3 Table — Table including baseline characteristics of patients treated with telaprevir and boceprevir, these patients are pooled in the primary analysis. (DOCX) [file pone.0161821.s004.docx]

**S3 Table. Baseline characteristics telaprevir and boceprevir treated patients**

| Characteristic | Telaprevir (n=265) | Boceprevir (n=202) | p-value  (TPR/BOC) |
| --- | --- | --- | --- |
| Age, y – mean (range) | 51 (19-71) | 51 (22-77) | 0.84 |
| Male sex – n (%) | 174 (66) | 145 (72) | 0.16 |
| White race – n (%)^a^ | 186 (89) | 135 (90) | 0.43 |
| HCV genotype – n (%)  Genotype 1 indeterminate  Genotype 1a  Genotype 1b | 31 (20)  121 (46)  91 (34) | 33 (16)  105 (52)  64 (32) | 0.35 |
| Previous response^b^  Naive  Relapse  Nonresponse  Viral breakthrough  Early discontinuation | 152 (59)  40 (15)  47 (18)  11 (4)  7 (3) | 121 (61)  36 (18)  31 (16)  5 (3)  4 (2) | 0.70 |
| Decompensated liver disease – n (%) | 12 (5) | 12 (6) | 0.49 |
| Metavir score F3-4^c^ | 96 (52) | 65 (52) | 0.97 |
| Laboratory values^d^ |  |  |  |
| Haemoglobin g/dl - mean (SD) | 9.1 (0.9) | 9.2 (0.9) | 0.29 |
| Leucocyte count x10^9^/L - mean (SD) | 6.8 (2.1) | 6.7 (2.2) | 0.74 |
| Neutrophil count x10^9^/L - mean (SD) | 3.5 (1.6) | 3.4 (1.5) | 0.66 |
| Platelet count x10^9^/L – mean (range) | 195 (51 – 764) | 188 (24-387) | 0.31 |
| Albumin g/l – mean (range) | 41.8 (24-51) | 40.9 (24-49) | 0.06 |
| Total bilirubin g/dl – median (IQR) | 10 (7-14) | 9.9 (7-14) | 0.82 |

^a^ Race: available in 360 patients; ^b^ Previous response: available in 454 patients (257 TPR, 197 BOC); ^c^Metavir score: available in 308 patients (184 TPR, 124 BOC); ^d^Lab values >10% missings in: neutrophil count, albumin;
