# Supplementary material for: Limited Generalizability of Registration Trials in Hepatitis C: A Nationwide Cohort Study
Source: PLoS One. 2016 Sep 6;11(9):e0161821. doi: 10.1371/journal.pone.0161821 (PMC5012685; doi:10.1371/journal.pone.0161821)
Supplement: S4 Table — Table showing effectiveness and safety results of telaprevir vs. boceprevir, these patients are pooled in the primary analysis. (DOCX) [file pone.0161821.s005.docx]

**S4 Table. Effectiveness and safety of telaprevir compared to boceprevir**

|  | **TPR (n=265)** | **BOC (n=202)** | **p-value** |
| --- | --- | --- | --- |
| **SVR on previous response, n (%)**  Naive (TPR n=152, BOC n=121)  Relapse (TPR n=40, BOC n=36)  Non response (TPR n=47, BOC n=31)  Viral breakthrough (TPR n=11, BOC n=5)  Early discontinuation (TPR n=7, BOC n=4)  Unknown (TPR n=8, BOC n=5) | 107 (70)  27 (69)  20 (43)  4 (36)  5 (71)  5 (63) | 84 (69)  26 (72)  13 (42)  1 (20)  1 (25)  3 (60) | 0.86  0.78  0.96  1.00*  0.24*  1.00* |
| **SVR on previous response, n (%)**  Naïve and Relapse (n=349)  Nonresponse,viral breakthrough, early  discontinuation (n=118) | 135 (70)  34 (47) | 110 (70)  18 (40) | 0.96  0.49 |
| **SAE on previous response, n (%)**  Naive (TPR n=152, BOC n=121)  Relapse (TPR n=40, BOC n=36)  Non response (TPR n=47, BOC n=31)  Viral breakthrough (TPR n=11, BOC n=5)  Early discontinuation (TPR n=7, BOC n=4)  Unknown (TPR n=8, BOC n=5) | 33 (22)  9 (23)  9 (19)  1 (9)  1 (14)  3 (38) | 18 (15)  4 (11)  5 (16)  3 (60)  1 (25)  1 (20) | 0.15  0.19  0.73  0.06*  1.00*  1.00* |
| **Mean sum AE previous response (±SD)**  Naive (TPR n=152, BOC n=121)  Relapse (TPR n=40, BOC n=36)  Non response (TPR n=47, BOC n=31)  Viral breakthrough (TPR n=11, BOC n=5)  Early discontinuation (TPR n=7, BOC n=4)  Unknown (TPR n=8, BOC n=5) | 3.0±2.2  2.7±1.8  3.0±2.5  1.9±2.1 2.7±0.8  3.0 ±1.5 | 2.8±2.3  3.4±2.2  2.7±2.9  3.6±3.2  2.8±1.0  3.2±2.1 | 0.63  0.10  0.82  0.36  0.58  0.83 |
| **Mean sum SAE previous response (±SD)**  Naive (TPR n=152, BOC n=121)  Relapse (TPR n=40, BOC n=36)  Non response (TPR n=47, BOC n=31)  Viral breakthrough (TPR n=11, BOC n=5)  Early discontinuation (TPR n=7, BOC n=4)  Unknown (TPR n=8, BOC n=5) | 0.3±0.7  0.3±0.5  0.2±.05  0.3±0.9 0.1±0.4  0.6±0.9 | 0.25±0.7  0.17±0.5  0.48±1.5  1.00±1.0  0.25±0.5  0.40±0.9 | 0.09  0.27  **0.02**  0.41  0.45  0.59 |

* analysis with Fisher exact as frequency counts <5
