# Supplementary material for: Limited Generalizability of Registration Trials in Hepatitis C: A Nationwide Cohort Study
Source: PLoS One. 2016 Sep 6;11(9):e0161821. doi: 10.1371/journal.pone.0161821 (PMC5012685; doi:10.1371/journal.pone.0161821)
Supplement: S5 Table — Table with categories of serious adverse events in eligible vs. ineligible patients. (DOCX) [file pone.0161821.s006.docx]

**S5 Table. Serious adverse events categories (number of events and number of patients)**

| Category^1^ | Eligible^2^  (37 SAEs) | Number of eligible patients (n=28) | Ineligible^3^  (103 SAEs) | Number of ineligible patients (n=60) | p-value  (Fisher exact) |
| --- | --- | --- | --- | --- | --- |
| Hepatobiliary | 1 (2.7) | 1 (3.6) | 23 (22.3) | 15 (25.0) | 0.017 |
| Anemia | 4 (10.8) | 3 (10.7) | 15 (14.6) | 14 (23.3) | 0.247 |
| Respiratory | 9 (24.3) | 8 (28.6) | 13 (12.6) | 10 (16.7) | 0.257 |
| Gastrointestinal | 5 (13.5) | 4 (14.3) | 9 (8.7) | 7 (11.7) | 0.738 |
| Circulatory | 0 (0.0) | 0 (0.0) | 8 (7.8) | 8 (13.3) | 0.051 |
| Psychiatric | 3 (8.1) | 3 (10.7) | 6 (5.8) | 5 (8.3) | 0.706 |
| General | 3 (8.1) | 3 (10.7) | 6 (5.8) | 6 (10.0) | 1.00 |
| Central nerve system | 0 (0.0) | 0 (0.0) | 5 (4.9) | 4 (6.7) | 0.302 |
| Endocrine | 1 (2.7) | 1 (3.6) | 4 (3.9) | 3 (5.0) | 1.00 |
| Musculoskeletal | 1 (2.7) | 1 (3.6) | 4 (3.9) | 3 (5.0) | 1.00 |
| Skin | 4 (10.8) | 3 (10.7) | 3 (2.9) | 3 (5.0) | 0.378 |
| Ear Nose Throat | 1 (2.7) | 1 (3.6) | 3 (2.9) | 2 (3.3) | 1.00 |
| Renal | 2 (5.4) | 2 (7.1) | 2 (1.9) | 2 (3.3) | 0.589 |
| Leucopenia | 0 (0.0) | 0 (0.0) | 1 (1.0) | 1 (1.7) | 1.00 |
| Pancytopenia | 1 (2.7) | 1 (3.6) | 0 (0.0) | 0 (0.0) | 0.318 |
| Nutritional | 0 (0.0) | 0 (0.0) | 1 (1.0) | 1 (1.7) | 1.00 |
| Reproductive system | 1 (2.7) | 1 (3.6) | 0 (0.0) | 0 (0.0) | 0.318 |
| Eye disorder | 1 (2.7) | 1 (3.6) | 0 (0.0) | (0 (0.0) | 0.318 |

^1^ Percentage is noted between brackets in whole table

^2^ One eligible patient died due to an accident

^3^ Seven ineligible patients died, causes: hepatic encephalopathy, decompensated liver disease and CVA, sepsis and CVA, CVA, renal insufficiency, endocarditis, and one patient died of unknown cause
