# Supplementary material for: Limited Generalizability of Registration Trials in Hepatitis C: A Nationwide Cohort Study
Source: PLoS One. 2016 Sep 6;11(9):e0161821. doi: 10.1371/journal.pone.0161821 (PMC5012685; doi:10.1371/journal.pone.0161821)
Supplement: S6 Table — Table showing outcomes of sensitivity analyses: analysis with most stringent criteria, analysis with strict exclusion of patients with co-morbidity, analysis without prohibited comedication as exclusion criterion. (DOCX) [file pone.0161821.s007.docx]

**S6 Table. Sensitivity analyses**

| **Sensitivity analysis** |  | **Eligible – n (%)** | **Ineligible – n (%)** | **p-value** |
| --- | --- | --- | --- | --- |
| Primary analysis | No of patients, n (%) | 247 (53) | 220 (47) |  |
|  | SVR (TN/relapse, n=348), n (%)  SVR (NR/other, n=118), n (%) | 137/186 (74)  29/60 (48) | 107/162 (66)  23/58 (40) | 0.12  0.34 |
|  | SAE, n (%) | 28 (11) | 60 (27) | <0.001 |
|  | Sum of SAEs – median (IQR) | 0 (0-0) | 0 (0-1) | 0.001* |
|  | AE, n (%) | 211 (85) | 199 (91) | 0.097 |
|  | Sum of AEs – median (IQR) | 2 (1-4) | 3 (1-5) | 0.039* |
| Sensitivity analysis: most strict analysis | No of patients, n (%) | 102 (22) | 365 (78) |  |
|  | SVR (TN/relapse, n=348), n (%)  SVR (NR/other, n=118), n (%) | 69/85 (81)  7/16 (44) | 175/263 (67)  45/102 (44) | 0.01  0.98 |
|  | SAE, n (%) | 12 (12) | 76 (21) | 0.04 |
|  | Sum of SAEs – median (IQR) | 0 (0-0) | 0 (0-0) | 0.034* |
|  | AE, n (%) | 84 (82) | 326 (89) | 0.06 |
|  | Sum of AEs – median (IQR) | 2 (1-4) | 3 (1-4) | 0.018* |
| Sensitivity analysis: more strict | No of patients, n (%) | 222 (48) | 245 (52) |  |
| exclusion of patients with co-morbidity | SVR (TN/relapse, n=348), n (%)  SVR (NR/other, n=118), n (%) | 127/167 (76)  26/54 (48) | 117/181 (65)  26/64 (41) | 0.02  0.41 |
|  | SAE, n (%) | 23 (10) | 65 (27) | <0.001 |
|  | Sum of SAEs – median (IQR) | 0 (0-0) | 0 (0-1) | <0.001* |
|  | AE, n (%) | 188 (85) | 222 (91) | 0.05 |
|  | Sum of AEs – median (IQR) | 2 (1-4) | 3 (1-5) | 0.001* |
| Sensitivity analysis: comedication not | No of patients, n (%) | 289 (62) | 178 (38) |  |
| included in analysis | SVR (TN/relapse, n=348), n (%)  SVR (NR/other, n=118), n (%) | 158/214 (75)  34/74 (46) | 86/134 (64)  18/44 (41) | 0.06  0.59 |
|  | SAE, n (%) | 38 (13) | 50 (28) | <0.001 |
|  | Sum of SAEs – median (IQR) | 0 (0-0) | 0 (0-1) | <0.001* |
|  | AE, n (%) | 253 (88) | 157 (88) | 0.83 |
|  | Sum of AEs – median (IQR) | 2 (1-4) | 3 (1-5) | 0.030* |

* analysis performed with Mann-Whitney U test

TN= Treatment Naïve; NR= Non responder; SAE= Serious Adverse Event; SVR= Sustained Virological Response; AE = Adverse Event
