# Supplementary material for: Limited Generalizability of Registration Trials in Hepatitis C: A Nationwide Cohort Study
Source: PLoS One. 2016 Sep 6;11(9):e0161821. doi: 10.1371/journal.pone.0161821 (PMC5012685; doi:10.1371/journal.pone.0161821)
Supplement: S7 Table — Table with exclusion criteria of new generation DAAs in comparison to our general criteria set. (DOCX) [file pone.0161821.s008.docx]

**S7 Table. Exclusion criteria of registration trials in new generation DAAs**

| **General exclusion criteria set of our study** | **Ledipasvir/sofosbuvir** | | **Paritaprevir/ritonavir, ombitasvir, dasabuvir** | |
| --- | --- | --- | --- | --- |
|  | **ION-1, ION-II** | **ION-III** | **Sapphire I, Pearl III, Pearl IV, Saphire II, Pearl II** | **Turquoise II (compensated cirrhotics)** |
| **Decompensated liver disease** | Clinical hepatic decompensation | Presence of cirrhosis | Presence of cirrhosis | CP-B, and -C cirrhosis |
| **Platelets < 90 x10^9^/L** | Platelets < 50 x10^9^/L | Platelets < 90 x10^9^/L | Platelets <120 x10^9^/L | Platelets <60 x10^9^/L |
| **Total bili > 1,8x ULN**† | Direct bili > 1.5x ULN† | Direct bili > ULN† | Indirect bili >1.5 ULN and direct bili > ULN† | Total bili ≥ 3.0 mg/dL |
| **Serum albumin < 3.3 g/dL** | Serum albumin < 3 g/dL | Serum albumin < 3 g/dL | Serum albumin < LLN† | Serum albumin < 2.8 g/dL |
| **Significant cardiac disease** | Significant cardiac disease | Significant cardiac disease | Significant cardiac disease | Significant cardiac disease |
| **Hb <12 g/dL for females or <13 g/dL for males** | Hb <11 g/dL for females and <12 g/dL for males | Hb <11 g/dL for females and <12 g/dL for males | Hb < LLN† | Hb < LLN† |
| **Active or recent malignancy** | Active or recent malignancy | Active or recent malignancy | Active or recent malignancy | Active or recent malignancy |
| **Absolute neutrophil count <1.2 x10^9^/L** | n/a | n/a | Absolute neutrophil count <1.5 x10^9^/L | Absolute neutrophil count <1.5 x10^9^/L |

† ULN = upper limit of normal; LLN = lower limit of normal; DAA = Direct Acting Antiviral
